# Corporate governance for sustainable development in Vietnam: Criteria for SOEs based on MCDM approach

**DOI:** 10.1371/journal.pone.0302306

**Published:** 2024-05-01

**Authors:** Phi-Dinh Hoang, Ly-Thi Nguyen, Binh-Quoc Tran, Dao-Thi Ta

**Affiliations:** Hanoi School of Business and Management, Vietnam National University, Hanoi, Vietnam; Istinye University: Istinye Universitesi, TURKEY

## Abstract

This research explores the nexus between corporate governance and sustainable development, focusing on State-Owned Enterprises (SOEs) in Vietnam. Recognizing the pivotal role of SOEs in the national economy, this study employs a Multi-Criteria Decision-Making approach (MCDM) to assess and enhance the corporate governance frameworks of these entities. First, the Data Envelopment Analysis (DEA) model is employed to identify the most qualified prospective SOEs firms based on several quantitative criteria. Then, the spherical fuzzy analytic hierarchy process (SF-AHP) model is used to identify priority weights for a given set of qualitative criteria, the Evaluation based on distance from average solution (EDAS) model is implemented to rank enterprises in the SOEs sector. To validate the proposed models, a case study conducted within the Vietnamese electric power industry is utilized. The MCDM methodology integrates diverse factors such as business management, corporate social responsibility, and corporate governance shareholder to construct a comprehensive evaluation framework. By applying this approach, the study aims to identify the key drivers and barriers influencing corporate governance practices within Vietnamese SOEs. The study’s findings illustrate the efficacy of the suggested approach in evaluating corporate governance performance, providing valuable insights for policymakers, corporate leaders, and stakeholders involved in shaping the governance landscape of SOEs in Vietnam. By aligning corporate governance with sustainable development principles, the research aims to contribute to the ongoing discourse on responsible business practices, offering practical recommendations to enhance the performance and resilience of SOEs in the pursuit of long-term socio-economic and environmental sustainability.

## 1. Introduction

Corporate governance (CG) plays a very important role in maintaining and developing a business. Corporate governance not only creates a reasonable corporate structure and organization consistent with the company’s characteristics and goals but also improves the labor efficiency of company employees while integrating company employees. At the same time, with corporate governance, the company can maintain, operate continuously without interruption, and develop.

In the current context of globalization and economic development, corporate governance is considered an important factor in ensuring sustainable development. In that context, Vietnam is no exception to this general trend, especially in applying corporate governance to promote sustainable development. With the increase in size and role of state-owned enterprises (SOEs), corporate governance has become a decisive factor in promoting sustainable development in Vietnam.

Many previous studies have mentioned the importance of corporate governance and sustainable development. Bahadori et al. [1] shows that businesses with good strategies and implementation on environmental, social and corporate governance factors often have better business performance and are more highly appreciated by the market. Tran et al. [2] and Sadiq et al. [3] finds the impacts of factors such as corporate policies, board of directors and management strategies on an organization’s ability to achieve sustainable goals and shows where policies and governance measures can be optimized to ensure sustainable development in ASEAN. While those studies provide an overview of the role of environmental, social and corporate governance factors in achieving sustainable development goals, further analysis may be needed on how to implement and measure the effectiveness of specific measures in each ASEAN member. As such, there are still many challenges that need to be overcome, especially in applying corporate governance standards and criteria into practice.

Sustainable development is characterized by its ability to fulfill the requirements of the current generation while safeguarding the capacity of future generations to do the same. It operates on a logical foundation that harmonizes economic advancement, social issue resolution, and environmental protection. In the context of business enterprises as a whole, sustainable development entails the implementation of business strategies and activities that concurrently safeguard, maintain, and augment the human and natural resources that will be indispensable in the future, while simultaneously satisfying the needs of the business and its present stakeholders, including customers, employees, and others [4]. Sustainable development is a strategic vision of the firm that aims to achieve long-term goals [5]. Businesses can pursue several strategies to attain this objective, including prioritizing gender equality, establishing social responsibility initiatives, managing environmental health and safety, and practicing responsible supply chain management [6].

While sustainable development solutions vary by industry, the fundamental principle of a sustainable business is to achieve a harmonious equilibrium among economic, social, and environmental progress. This balance requirement must guide the implementation of business strategies and operations to guarantee not only economic efficiency but also the management of social risks and environmental health and safety. At present, an extensive array of criteria exists for assessing the sustainable development of enterprises. The international and Vietnamese criterion sets are both qualitative and quantitative, and they evaluate businesses according to the following three factors: economic, social, and environmental. Organizations may employ this collection of standards to assess the degree of sustainable development their operations have achieved, enabling them to adapt their strategies to align with both domestic and global development trends.

Although corporate governance and sustainable development have become important topics in business research, significant gaps still exist that require detailed attention and research from the research and business communities. One of the important gaps is the combination of corporate governance and sustainable development. Currently, many businesses are facing pressure from both the community and investors to integrate sustainable development strategies into their management strategies. However, there are still many issues that require extensive research on how to make these two fields interact and enhance each other. Specifically, how effective management can contribute to the formulation and maintenance of sustainable development strategies is an area that has not been fully explored. Another gap lies in the level of integration between corporate management and the challenges of the global market. Despite efforts to find a balance between competitiveness and social responsibility, much remains to be done to better understand how corporate governance can meet market demands. globally while maintaining a commitment to sustainable development. Additionally, evaluating and measuring performance on the sustainability side of corporate governance is another area that needs focus. How to quantify the contribution of corporate governance to the environment, society, and economy remains a major challenge awaiting detailed research. In summary, the need for research on the interaction between corporate governance and sustainable development, as well as how to evaluate and measure performance in this area, remain important aspects that need to be addressed clearly to guide the sustainable development of business organizations in the future.

State-owned enterprises (SOEs) in Vietnam have unique characteristics compared to private enterprises and foreign-invested enterprises. Firstly, SOEs are usually organizations managed and owned by the Government. Major decisions such as business strategies, investments and other important decisions are often decided or directly intervened in by the Government. Then, the main goal of SOEs is often to serve national and social interests. They often contribute to economic development, create employment opportunities, provide basic services, and have great social responsibility in many strategic sectors such as energy, telecommunications, transportation, banking, state-owned enterprises account for a large proportion of the market and can ensure social security. However, SOEs’ business decisions are often subject to intervention from regulatory agencies and the Government. This can reduce management flexibility and quick response to the market.

Sustainable development is important for state-owned enterprises because it not only meets social responsibility and environmental protection but also brings business benefits and long-term opportunities. The implementation of sustainable development is a key factor in building and maintaining their success in an era of increasing sensitivity to social and environmental issues. Although assessing the sustainable development of businesses is important, there are also some shortcomings and challenges related to the current set of assessment criteria in Vietnam. For instance, there is a lack of uniformity and standardization in sustainable development assessment criteria [7]. Businesses often face having to comply with many different standards, making management and reporting difficult. Moreover, existing criteria sets often struggle to measure a business’s actual performance against sustainability goals. Some indicators may be too late or do not reflect the true reality of the business. Most current evaluation criteria focus on imposing requirements and regulations that do little to stimulate and encourage businesses to take sustainable actions from a conscious and intrinsic motivation. To address the research gaps presented above, this study is designed to identify criteria for evaluating the effectiveness of sustainable development-oriented corporate governance for state-owned enterprises Vietnam. By integrating, combining and selecting criteria in the ESG model and synthesizing criteria in the sustainable competitiveness pyramid by Hoang [8], this study also highlights the importance of these factors for with investors in making investment decisions. Corporate governance oriented towards sustainable development has become a major social issue internationally and domestically. Many investors are looking for companies that fit their evaluation criteria, and regulators in many countries are introducing new regulations or laws. Consulting firms and private organizations are distributing corporate governance models (such as ESG) that also reflect some country-specific characteristics. However, up to now there has not been a complete, universal set of criteria agreed upon by relevant stakeholders and proven by academic research for large state-invested enterprises, for state-owned enterprises in Vietnam. This study proposes MCDM model that includes evaluation criteria specific to Vietnam’s state-owned enterprises and ranking potential enterprises.

MCDM is an essential concept that helps evaluate several options for a decision-making problem by taking into account multiple dimensions and indications that may contradict each other [9]. The main goal of the MCDM method is to determine the best choice among a range of possibilities. To achieve this goal, traditional and fuzzy MCDM procedures have been used. Fuzzy models have been found to be more effective in dealing with ambiguity in human opinions, leading to more realistic and practical conclusions [10]. Selecting the best suitable SOEs firms is clearly MCDM issue. This is because there are multiple conflicting characteristics, such as business management, corporate social responsibility, and corporate governance shareholder factors, that need to be considered when assessing different SOEs organizations. An in-depth evaluation of these aspects is essential to support efficient decision-making in this situation. Furthermore, the assessment of the performance of decision-making units (DMUs) that perform transformations on numerous inputs and outputs is the focus of DEA. The outcomes of DEA offer an efficiency metric for every DMU, enabling the differentiation of efficient and inefficient DMUs and the identification of the efficient peers of each inefficient DMU [11]. As a result of the formal analogies between DEA and MCDM and the efficacy of DEA in performance evaluation, some authors have suggested employing DEA as a tool for MCDM [12]. Recent publications that have initiated an examination of the correlation between DEA and MCDM demonstrate the potential utility of DEA in the context of MCDM. For examples, Selamzade et al. [13] employed the DEA approach in conjunction with MCDM methodologies to assess the efficiency of OECD countries. Yilmaz [14] utilized DEA and Fuzzy complex proportional assessment (Fuzzy COPRAS) to assess the effectiveness of 11 wind power stations situated in Turkey’s Marmara Region.,

In this study, the DEA methodology is used to find the most proficient potential State-Owned Enterprises (SOEs) companies in the first stage. Then, the SF-AHP method is utilized to determine the priority weights of a given set of criteria, which is then followed by the application of the EDAS model to rank enterprises in the SOEs sector. The proposed models are validated using a genuine case study conducted in the Vietnamese electric power industry. The findings of this study can be used in corporate governance practices, investors’ investment decisions, and management policy formulation by government agencies. This study hopes to contribute to adjusting and improving policies and regulations on corporate governance in Vietnam, thereby ensuring that corporate governance is not only an important tool to increase enhancing business performance but also being a decisive factor in promoting the sustainable development of the economy.

The remaining sections of this study are structured as follows. The next section provides a thorough evaluation of the existing literature pertaining to criteria and models. Section 3 examines the techniques employed to ascertain the weights and rankings of alternatives. A case study is given in Section 4. The analysis of the findings is presented and discussed in Section 5. Section 6 examines conclusions, limitations and future works.

## 2. Literature review

### 2.1 Literature review for criteria

CG is defined in a number of previous studies. Shleifer and Vishny [15] define that corporate governance is a system of principles, regulations, mechanisms and management practices established and implemented to operate and control the activities of a business organization, especially joint stock companies, to ensure transparency, accountability, fairness, and respect for the rights of shareholders and other stakeholders. OECD [16] defines that corporate governance encompasses the interconnected connections among a company’s management, board of directors, shareholders, and other stakeholders. Additionally, it establishes the framework for defining the company’s goals and determining the methods for achieving those goals, as well as monitoring success. Other definitions of the concept are shown in Table 1.

**Table 1:** Corporate governance definitions in previous studies.

Ly-Thi Nguyen	Ly-Thi Nguyen
Cadbury [17]	Corporate governance refers to the framework and processes through which companies are managed and overseen. Boards of directors have the responsibility of governing their companies. The shareholders’ responsibility in governance is to select the directors and auditors and ensure that a suitable governance framework is established.
Brennan and Solomon [18]	The system of checks and balances, encompassing both internal and external mechanisms, ensures that corporations fulfill their obligation to all stakeholders and conduct their commercial activities in a socially responsible manner across all domains.
Cannon [19]	Enterprise governance encompasses the collective actions involved in internally regulating a business to ensure compliance with legal requirements, as well as the responsibilities imposed on the company by its ownership and management. The process involves the administration of assets, including their management, and utilization.
Demirag [20]	Corporate governance refers to the framework through which firms are managed, supervised, and held responsible to shareholders and other stakeholders. Control encompasses both direct and indirect pressures from financial markets.

In the setting of state-owned enterprises, Thuy et al. [21] evaluate the impact of corporate governance factors on the implementation of corporate social responsibility in Vietnam. The research focuses on the role of state management, especially state ownership, and how to regulate the relationship between corporate governance and corporate social responsibility. The results of the study provide insight into how corporate governance structures and state management factors can influence corporate behavior and commitment to social responsibility. The study of Chigudu [22] provides insight into how the implementation of corporate governance principles can influence the performance and sustainability of state-owned enterprises in South Africa.

Several studies focus on identifying which internal corporate governance mechanisms promote corporate sustainability [23, 24]. The summary of this research paper includes analyzing the relationship between internal governance factors such as board structure, authorization, internal control, and resource regeneration with sustainable performance. sustainability of the business. Nevertheless, the research just pay attention on some specific internal governance mechanisms without covering all governance factors that can affect corporate sustainability. Our study will fill that research gap.

**Fig 1:**
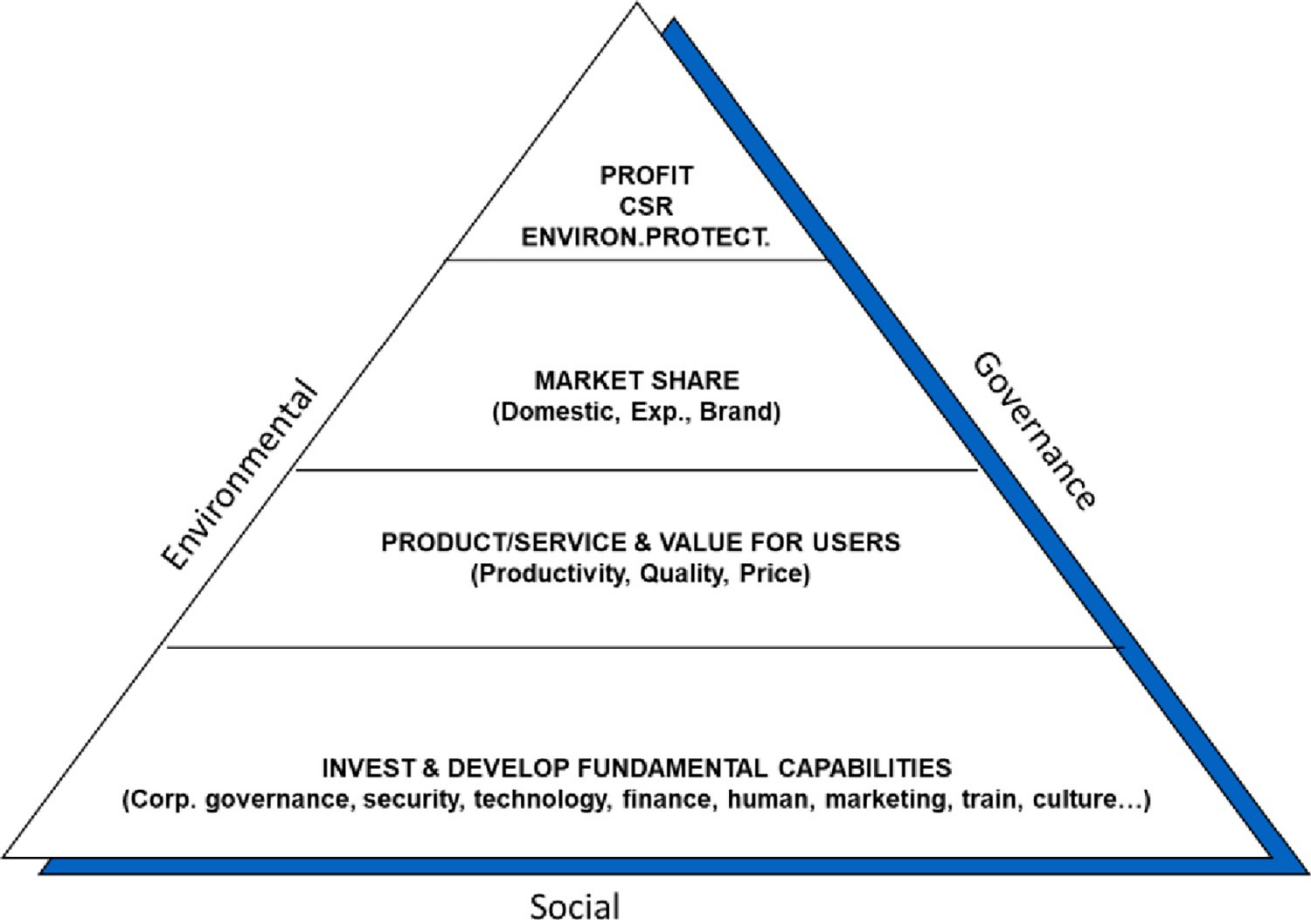
Integration of ESG and sustainable competitiveness of business. Source: adapted and modified by the authors of this study.

The pyramid of sustainable competitiveness of businesses (Fig 1) was introduced in 2014 by Hoang [8]. This is a model that not only shows the balanced integration between the three main factors of environment, social, and governance (ESG—the three sides of the pyramid), but also demonstrates the steps and measures those businesses need to implement to achieve the goal of sustainable competitiveness (four floors inside the tower).

The bottom level—the first level—of the pyramid shows us the basic elements that each business needs to perform well before achieving further results. Specifically:

Technology and innovation governance: the management and investment in research and development (R&D) facilitate the development of sustainable processes and technologies. Urban et al. [25] argue that corporate-level governance, collaboration/technology transfer have shaped leading wind energy technologies in China and to a lesser extent in India. The study concludes that technological cooperation between China, India and Europe has become more multifaceted and increasingly South-led. Another example that confirms the importance of investing in technological innovation for sustainable development at the corporate level is that companies such as Tesla (USA), or Vinfast (Vietnam) have invested in R&D to develop electric vehicles and sustainable energy solutions, contributing to the transition to a low-carbon economy. Likewise, having financial capital allows businesses to invest in renewable energy sources, energy-saving technologies, and sustainable production processes, leading to reduced environmental impacts [26]. For example, companies like Interface Inc. has allocated financial resources to develop and implement sustainable production practices, leading to reduced waste, energy use and greenhouse gas emissions [27].

Risk management and enterprise security: The non-traditional security management equation by Hoang et al. [28] affirms that risk management and enterprise security are one of the important factors determining the development of enterprises. sustainable development of a business. Financial capital assists businesses in managing sustainability-related risks, such as the impacts of climate change and supply chain disruptions [29]. As evidence, companies like Unilever have integrated financial risk assessments into their sustainable development strategies, helping them identify and address risks related to water scarcity, climate change and resource availability [30].

Also at this level, human resource management also plays a key role in the sustainable development of the business. This has been proven by previous studies. Specifically, Christmann and Taylor [31] argue that human resource management plays an important role in providing sustainable education and training for employees, equipping them with the necessary knowledge and skills to contribute to sustainable development. In fact, it is proven that companies like Interface Inc. has invested in employee training programs to raise awareness of sustainability issues and encourage awareness and action on sustainability goals at all levels of the organization [27]. Delmas and Toffel [32] argue that effective implementation of human resource management promotes employee engagement and participation in sustainability initiatives, which contributes to improved sustainability performance. As evidence, companies like Google have implemented initiatives that empower employees to contribute ideas and initiatives for environmental sustainability, leading to positive environmental outcomes [33].

The next level of the business’s sustainable competitiveness pyramid shows that after the business has invested in the basic resources in the first level, at this level the business will achieve some initial successes such as: can produce quality products and services to sell to the market and create value for customers. Then, the next layer shows that, once developed to a certain level, the business will achieve a certain market share through developing the domestic market, exporting, and building a brand. Many studies have been providing insight into the role of brand and reputation in promoting sustainable development at the corporate level [34]. These studies highlight the importance of brand image, reputation and CSR (Corporate social responsibility) initiatives in driving stakeholder engagement, differentiation, and investor confidence., risk mitigation, employee engagement and advocacy for sustainable practices.

Some case studies can demonstrate what has been stated above, for examples, Bhattacharya et al. [35] emphasize that mutually beneficial CSR initiatives strengthen the relationship between stakeholders and the company and contribute to sustainable development. The author also asserts that a strong brand and reputation for sustainability can promote stakeholder engagement, leading to collaborative efforts toward sustainable development. Linnenluecke et al. [33] highlight that sustainability reporting and a positive reputation related to sustainability can enhance a company’s performance and differentiation in the market. Thus, a sustainable brand can have a distinct competitive advantage over its competitors, providing a sustainable competitive advantage and good positioning in the market. Additionally, a reputable sustainability brand can attract socially responsible investors and increase access to capital [36].

Fig 1 shows that the top of the pyramid is the highest goal that a business wants to achieve when implementing a sustainable development strategy, which is profit, practicing social responsibility, and protecting the environment. The ultimate goal of sustainable development at the corporate level is often described as creating long-term value for all stakeholders. This perspective is consistent with stakeholder theory, which emphasizes that businesses should consider the interests of all stakeholders, not just shareholders. Friedman [37] expressed a contrasting view focusing solely on profit maximization, but it is important to note that contemporary research and thinking has evolved to emphasize the broader purpose of development. sustainable development. Elkington [38] introduced the concept of the triple bottom line, which combines economic, social and environmental aspects of sustainable development. This approach recognizes that companies have a responsibility to create value not only for shareholders but also for society and the environment. Additionally, Carroll [39] introduced the concept of the "corporate social responsibility pyramid", which highlights the moral and ethical obligations of business to its stakeholders. The pyramid includes economic, legal, ethical, and philanthropic responsibilities, emphasizing the need for companies to go beyond financial performance and engage in sustainable practices.

Research on the importance of business management effectiveness in enterprises has attracted great attention from the research community thus creating a field rich with diverse perspectives and research methods. Some studies such as the work of Barney [40] and Mintzberg [41] focused on the importance of business strategy and how it affects business performance. These studies often identify strategy as a key factor in business management that has a profound impact on the level of success.

Corporate governance for sustainable development for state-owned enterprises requires consideration of all aspects of business operations, including economic, environmental, and social. This is an important direction to ensure that state-owned enterprises contribute to the country’s sustainable development and meet global challenges related to climate change, minimizing impacts on the environment, and build a fair and prosperous society.

From the above discussion, this study synthesizes and balances all above-mentioned factors, analyzes their importance in ensuring sustainable development, and proposes three main criteria of corporate governance for sustainable development for state-owned enterprises in Vietnam, including: Business management, CSR, corporate governance shareholder.

### 2.2. Literature review for MCDM models

The DEA technique is commonly utilized to evaluate the effectiveness of DMUs. DEA analysis was presented by Charnes, Cooper, and Rhodes (CCR) in 1978 [42]. The CCR technique used a non-parametric approach to create a production possibility frontier curve using DMU data collecting. The DMUs’ effectiveness was determined and compared using different mathematical programming methods. In 1984, Banker, Charnes, and Cooper (BCC) expanded the concept of CCR by computing variable returns to scale (VRS) scenarios. The BCC model that was developed allowed for a more thorough analysis of DMU efficiency [43]. Tone proposed a measure of efficiency called slacks-based measure (SBM) that includes the excess input and deficit of output in the units’ aim function. The SBM model is non-radial, meaning its inputs and outputs do not need to be verified simultaneously [44]. In contrast, the EBM model considers both radial and non-radial aspects, enabling a more accurate efficiency assessment. The EBM model precisely represents the unit’s efficiency being assessed and the distinction between its radial and non-radial components [45]. The outcomes of DEA yield an efficiency measure for each DMU, enabling the distinction between efficient and inefficient DMUs, as well as the identification of efficient peers for each inefficient DMU. The efficacy of DEA in assessing performance, along with the formal similarities between DEA and MCDM (which become apparent when DMU is substituted with alternatives, outputs are substituted with criteria to be maximized, inputs are substituted with criteria to be minimized, and so forth), has led certain authors to suggest employing DEA as a tool for MCDM [12]. Several recent publications have started examining the correlation between DEA and MCDM demonstrating the potential value of DEA in MCDM. For instance, Mousavi-Nasab and Sotoudeh-Anvari [46] investigated the application of DEA as a tool for material selection in MCDM. DEA can be utilized to address this issue, specifically when taking into account a conventional observation. However, DEA is not capable of substituting MCDM in this field as a whole.

The Analytic Hierarchy Process (AHP) is a highly effective methodology for Multiple Criteria Decision Making (MCDM). It addresses the challenges posed by complicated problems with multiple criteria by employing hierarchical structures and pairwise comparison matrices. This process typically comprises the subsequent steps: The process involves constructing AHP framework to organize the objectives, criteria, and alternatives in a hierarchical structure. This is followed by creating a pairwise comparison decision matrix to assess the relative importance of the criteria. The next step is to calculate the weights of the criteria and finally, test the consistency of the results [47]. The consistency ratio is a crucial metric in the AHP technique. It is used to assess the consistency of the pairwise comparison replies. The author suggests that for a problem to be effectively constructed, it is advisable to have a consistency ratio of 0.1 or below. While AHP depends on the assessments made by decision makers (DMs) to establish priority scales, it acknowledges that the judgments of DMs may lack consistency. In such instances, AHP aims to quantify the level of inconsistency and enhance the judgments made by DMs. The priority vector in the AHP model is a numerical ranking of alternatives that expresses a preference order between them. The principal eigenvector represents the priority vector of a consistent matrix. In the case of a positive reciprocal pairwise comparison matrix that allows for inconsistency, the principal eigenvector is necessary to represent the priorities associated with that matrix, If the Inconsistency is within a specified threshold.

Standard fuzzy sets contain multiple expansions, and the most notable ones can be summarized as follows: Type-1 intuitionistic fuzzy sets, as proposed by Atanassov [48] incorporate both membership and non-membership degrees to represent the degree to which an element belongs or does not belong to a fuzzy set. Unlike other models, Type-1 intuitionistic fuzzy sets do not explicitly express hesitancy degree independently. However, the hesitancy degree may still be determined as the complement of the sum of the membership and non-membership degrees, which always adds up to one. Subsequently, Type-2 intuitionistic fuzzy sets were formulated to encompass a wider range of membership and non-membership degrees, ensuring that the total of their squares is not greater than one. Neutrosophic sets, introduced by Smarandache [49], are an expanded form of Intuitionistic fuzzy sets. Contrary to Intuitionistic fuzzy sets, every element in a neutrosophic set possesses a specific level of truthfulness, uncertainty, and falsehood. These parameters can be interpreted as degrees of membership, non-membership, and hesitancy, respectively. Hesitant fuzzy sets, as proposed by Torra [50], were designed to handle scenarios where many membership functions are seen as potential options. Picture fuzzy sets, as discussed by Cuong and Kreinovich [51], are seen as an expanded form of intuitionistic fuzzy sets. The attitudes of voters, such as abstention, no, and refusal, can be represented as positive, neutral, negative, and refusal membership degrees in a picture fuzzy set manner. The q-rung orthopair fuzzy sets offer more advantages compared to intuitionistic fuzzy sets since they provide a larger range for expressing opinions on membership and non-membership degrees. However, these sets do not allow for independent modeling of the degree of reluctance.

The concept of spherical fuzzy sets, Introduced by Kutlu Gündoğdu and Kahraman [52], is based on a three-dimensional spherical geometry. These sets are constructed utilizing the idea of type-2 intuitionistic fuzzy sets and neutrosophic sets. In the context of spherical fuzzy sets, the spherical fuzzy is conceptualized as a volume rather than a solid. This enables us to allocate membership, non-membership, and hesitation parameters separately and within a more expansive domain. Geometric depictions of spherical fuzzy sets, intuitionistic fuzzy sets, neutrosophic sets, and type-2 intuitionistic fuzzy sets. In addition, spherical fuzzy sets are utilized in hybrid MCDMs that are merged with the AHP approach. Jaller and Otay [53] addressed the issue of determining the optimal site for an oil station using the SF-AHP and Weighted Aggregated Sum Product Assessment (WASPAS) model. Mathew et al. [54] addressed the issue of selecting a manufacturing system and devised an AHP TOPSIS approach specifically for this task. Nguyen et al. [55] employed an innovative approach by combining DEA with SF-AHP and WASPAS to identify sustainable suppliers in the steel industry.

The Evaluation based on distance from average solution (EDAS) is a novel and effective MCDM methodology [56]. It assesses the attractiveness of alternatives by calculating the total distance between each alternative and its corresponding average for each criterion. Contrary to TOPSIS and VIKOR, which rely on the concept of proximity to ideal solutions, EDAS technique use the average point as a benchmark. It employs two metrics, specifically the positive distance from the average and the negative distance from the average, to establish the ranking of alternatives. The EDAS approach has lately been used with numerous fuzzy extensions in diverse applications. Turskis et al. [57] employed precise numerical values and included AHP, EDAS methods to evaluate priority cultural heritage structures. Other authors have utilized triangular fuzzy numbers to create an AHP and EDAS model for many areas, including organization strategy formulation [58], supplier evaluation [59].

While numerous recent studies have examined various issues and proposed MCDM-based solutions to address them, there is currently no research that offers a solution to assess the corporate governance for sustainable development problem specifically focusing on criteria for State-Owned Enterprises (SOEs) in Vietnam using MCDM model. Hence, this study seeks to address the existing gap. Initially, the DEA methodology is used to find the most proficient potential SOEs companies. Subsequently, the SF-AHP method is utilized to determine the priority weights of a given set of criteria, which is then followed by the application of the EDAS model to rank enterprises in the SOEs sector. The proposed models are validated using a genuine case study conducted in the Vietnamese electric power industry.

## 3. Methodology

### 3.1. DEA Models-Preliminaries

This part presents a succinct mathematical framework for DEA comprising the Banker–Charnes–Cooper model (BCC), Charnes–Cooper–Rhodes model (CCR), Slacks–Based Measure model (SBM), and Epsilon–Based Measure model (EBM). The following is a description of the symbols and annotations that were employed in the model:

n: number of decision-making units (DMUs)

DMUi: the i-th DMU, *i* = 1,2,I,*n*

DMU0: the DMU target

*a*_0_ = (*a*_01_, *a*_02_,…,*a*_0*p*_): input vector of DMU0

*b*_0_ = (*b*_01_, *b*_02_,…,*b*_0*q*_): output vector of DMU0

*a*_*i*_ = (*a*_*i*1_, *a*_*i*2_,…,*a*_*ip*_): input vector of DMUi, *i* = 1,2,I,*n*

*b*_*i*_ = (*b*_*i*1_, *b*_*i*2_,…,*b*_*iq*_): output vector of DMUi, *i* = 1,2,I,*n*

*u*∈*R*^*p*×1^: weight-input vector

*v*∈*R*^*q*×1^: weight-output vector

#### 3.1.1. Charnes–Cooper–Rhodes model (CCR)

The CCR model was initially proposed by Charnes et al. [42]. The multiplier model of the CCR input-oriented (CCR-I) is defined by Eq (1).


$$\underset{u,v}{\mathrm{Max}}\xi =\frac{v^{T}b_{0}}{u^{T}a_{0}}$$

Subject to:

$$v^{T}b_{e}\leq u^{T}a_{e},e=1,2,\ldots ,n$$

u≥0,v≥0 (1)

#### 3.1.2. Banker–Charnes–Cooper model (BCC)

Wen [60] pioneered the BBC input-oriented (BBC-I) methodology. Eq (2) describes the model of BBC-I in a linear framework.


$$\underset{u,v}{\mathrm{Max}}\xi =\frac{v^{T}b_{0}-v_{0}}{u^{T}a_{0}}$$

Subject to:

$$\frac{v^{T}b_{e}-v_{0}}{u^{T}a_{e}}\leq 1,e=1,2,\ldots ,n$$

u≥0,v≥0 (2)

#### 3.1.3. Slacks-Based Measure model (SBM)

The SBM model was initially proposed by Farrell [61]. Based on the assumption of constant returns-to-scale, the input-oriented approach of SBM is referred to as SBM-I-C. As evident from Eq (3), the linear model is represented by Eq (3):

$$\omega_{In}^{*}=\underset{\alpha ,s^{-},s^{+}}{\mathrm{Min}}1-\frac{1}{p}\sum_{i=1}^{p}\frac{s_{i}^{-}}{a_{i0}}$$

Subject to:

$$\sum_{e=1}^{n}a_{ie}\alpha_{e}=a_{i0}-s_{i}^{-},i=1,2,\ldots ,p$$

$$\sum_{e=1}^{n}b_{re}\alpha_{e}=b_{r0}+s_{r}^{+},r=1,2,\ldots ,q$$

$$\alpha_{e}\geq 0,e=1,2,\ldots ,n$$

$$s_{i}^{-}\geq 0,i=1,2,\ldots ,p$$

$$s_{r}^{+}\geq 0,r=1,2,\ldots ,q$$ (3)

#### 3.1.4. Epsilon-Based Measure model (EBM)

Tone and Tsutsui [62] introduced the EBM model as a means to address the limitations observed in the CCR and SBM models. There are *n* DMUs (*j* = 1,2,I,*n*) in the EBM model, with *m* inputs (*i* = 1,2,I,*m*) and *s* outputs (*r* = 1,2,I,*s*). $X=\{x_{ij}\}\in R^{m\times n}$ and $Y=\{y_{rj}\}\in R^{s\times n}$, the input and output matrices are defined as matrices X and Y, respectively, where X and Y are matrices that contain only non-negative values. As depicted in Eq (4), the input-oriented model with a continuous return to scale (EBM-I-C) is illustrated.


$$\delta^{*}=\underset{\theta ,\lambda ,s^{-}}{\mathrm{Min}}\theta -\varepsilon_{x}\sum_{i=1}^{m}\frac{{w_{i}^{-}s}_{i}^{-}}{x_{io}}$$

Subject to

$$\sum_{j=1}^{n}x_{ij}\lambda_{j}=\theta x_{io}-s_{i}^{-},i=1,..,m$$

$$\sum_{j=1}^{n}y_{rj}\lambda_{j}\geq y_{ro},r=1,..,s$$

$$\lambda_{j}\geq 0,j=1,2,\ldots ,n$$

$$s_{i}^{-}\geq 0,i=1,2,\ldots ,m$$ (4)

where *λ*_*j*_ refers to the intensity vector of the DMU, the subscript “*o*” indicates that the DMU is under evaluation, the terms $s_{i}^{-}$ and $w_{i}^{-}$ reflect the slack and weight associated with the *i*^*th*^ input, a parameter *ε*_*x*_ represents the dispersion of the inputs, while and *θ* represents the radial properties.

### 3.2. Spherical fuzzy Sets-Preliminaries

Intuitionistic and Pythagorean fuzzy membership functions encompass parameters for membership, non-membership, and hesitancy, which can be ascertained using $\gamma_{{\tilde{F}}_{S}}=1–\alpha_{{\tilde{F}}_{S}}-\beta_{{\tilde{F}}_{S}}$ or $\gamma_{{\tilde{F}}_{S}}=1-\sqrt{1-\alpha_{{\tilde{F}}_{S}}^{2}-\beta_{{\tilde{F}}_{S}}^{2}}$, respectively. Spherical fuzzy sets [63] provide decision-makers with the capability to attach hesitancies to decisions pertaining to a broader domain in an independent way. This is achieved through the utilization of spherical fuzzy sets.

Definition 1. The denotation of the Spherical fuzzy set ${\tilde{F}}_{S}$ of the universe *X* is as follows.

$${\tilde{F}}_{S}=\{x,(\alpha_{{\tilde{F}}_{S}}(x),\beta_{{\tilde{F}}_{S}}(x),\gamma_{{\tilde{F}}_{S}}(x))|x\in X\}$$ (5)

$$\alpha_{{\tilde{F}}_{S}}(x):X\to [0,1],\beta_{{\tilde{F}}_{S}}(x):X\to [0,1],\gamma_{{\tilde{F}}_{S}}(x):X\to [0,1]$$

$$0\leq \alpha_{{\tilde{F}}_{S}}^{2}(x)+\beta_{{\tilde{F}}_{S}}^{2}(x)+\gamma_{{\tilde{F}}_{S}}^{2}(x)\leq 1$$ (6)

and with ∀*x*∈*X*, for each *x*, $\alpha_{{\tilde{F}}_{S}}(x)$ for membership, $\beta_{{\tilde{F}}_{S}}(x)$ for non−membership, and $\gamma_{{\tilde{F}}_{S}}(x)$ for hesitancy levels of *x* to ${\tilde{F}}_{S}$.

Definition 2. The six fundamental operations of the SFS are outlined as follows.

Union operation

$${\tilde{F}}_{S}\cup {\tilde{E}}_{S}={\{max\{\alpha }_{{\tilde{F}}_{S}},\alpha_{{\tilde{E}}_{S}}\},min{\{\beta }_{{\tilde{F}}_{S}},\beta_{{\tilde{E}}_{S}}\},min\{(1-{{\left((max\{\alpha_{{\tilde{F}}_{S}},\alpha_{{\tilde{E}}_{S}}\}\right)}^{2}+{\left(min{\{\beta }_{{\tilde{F}}_{S}},\beta_{{\tilde{E}}_{S}}\}\right)}^{2}))}^{0.5},max\{\gamma_{{\tilde{F}}_{S}},\gamma_{{\tilde{E}}_{S}}\}\}\}$$ (7)

Intersection operation

$${\tilde{F}}_{S}\cap {\tilde{E}}_{S}={\{min\{\alpha }_{{\tilde{F}}_{S}},\alpha_{{\tilde{E}}_{S}}\},max{\{\beta }_{{\tilde{F}}_{S}},\beta_{{\tilde{E}}_{S}}\},max\{(1-{{\left((min\{\alpha_{{\tilde{F}}_{S}},\alpha_{{\tilde{E}}_{S}}\}\right)}^{2}+{\left(max{\{\beta }_{{\tilde{F}}_{S}},\beta_{{\tilde{E}}_{S}}\}\right)}^{2}))}^{0.5},min\{\gamma_{{\tilde{F}}_{S}},\gamma_{{\tilde{E}}_{S}}\}\}\}$$ (8)

Addition operation

$${\tilde{F}}_{S}⊕{\tilde{E}}_{S}=\{\sqrt{{(\alpha }_{{\tilde{F}}_{S}}^{2}+\alpha_{{\tilde{E}}_{S}}^{2}–\alpha_{{\tilde{F}}_{S}}^{2}\alpha_{{\tilde{E}}_{S}}^{2})},\beta_{{\tilde{F}}_{S}}\beta_{{\tilde{E}}_{S}},\sqrt{\left(1-\alpha_{{\tilde{E}}_{S}}^{2})\gamma_{{\tilde{F}}_{S}}^{2}+(1-\alpha_{{\tilde{F}}_{S}}^{2})\gamma_{{\tilde{E}}_{S}}^{2}–\gamma_{{\tilde{F}}_{S}}^{2}\gamma_{{\tilde{E}}_{S}}^{2}\right\}}\}$$ ((9))

Multiplication operation

$${\tilde{F}}_{S}⊗{\tilde{E}}_{S}=\{\alpha_{{\tilde{F}}_{S}}\alpha_{{\tilde{E}}_{S}},\sqrt{\left(\beta_{{\tilde{F}}_{S}}^{2}+\beta_{{\tilde{E}}_{S}}^{2}–\beta_{{\tilde{F}}_{S}}^{2}\beta_{{\tilde{E}}_{S}}^{2}\right)},\sqrt{(1-\beta_{{\tilde{E}}_{S}}^{2})\gamma_{{\tilde{F}}_{S}}^{2}+(1-\beta_{{\tilde{F}}_{S}}^{2})\gamma_{{\tilde{E}}_{S}}^{2}–\gamma_{{\tilde{F}}_{S}}^{2}\gamma_{{\tilde{E}}_{S}}^{2}}\}$$ (10)

Multiplication by a scalar; *σ*>0

$$\sigma .{\tilde{F}}_{S}=\{\sqrt{{1-(1-\alpha_{{\tilde{F}}_{S}}^{2})}^{\sigma }},\beta_{{\tilde{F}}_{S}}^{\sigma },\sqrt{{\left(1-\alpha_{{\tilde{F}}_{S}}^{2}\right)}^{\sigma }-{\left(1-\alpha_{{\tilde{F}}_{S}}^{2}-\gamma_{{\tilde{F}}_{S}}^{2}\right)}^{\sigma }}\}$$ (11)

Power of F_*S*_; *σ*>0

$${\tilde{F}}_{S}^{\sigma }=\{\alpha_{{\tilde{F}}_{S}}^{\sigma },\sqrt{1-{\left(1-\beta_{{\tilde{F}}_{S}}^{2}\right)}^{\sigma }},\sqrt{({1-\beta_{{\tilde{F}}_{S}}^{2})}^{\sigma }-{\left(1-\beta_{F_{S}}^{2}-\gamma_{{\tilde{F}}_{S}}^{2}\right)}^{\sigma }}\}$$ (12)

Definition 3. For these SFSs ${\tilde{F}}_{S}=(\alpha_{{\tilde{F}}_{S}},\beta_{{\tilde{F}}_{S}},\gamma_{{\tilde{F}}_{S}})$ and ${\tilde{E}}_{S}=(\alpha_{{\tilde{E}}_{S}},\beta_{{\tilde{E}}_{S}},\gamma_{{\tilde{E}}_{S}})$, the followings are valid under the condition *σ*, *σ*_1_, *σ*_2_>0.


$${\tilde{F}}_{S}⊕{\tilde{E}}_{S}={\tilde{E}}_{S}⊕{\tilde{F}}_{S}$$ (13)

$${\tilde{F}}_{S}⊗{\tilde{E}}_{S}={\tilde{E}}_{S}⊗{\tilde{F}}_{S}$$ (14)

$$\sigma ({\tilde{F}}_{S}⊕{\tilde{E}}_{S})=\sigma {\tilde{F}}_{S}⊕\sigma {\tilde{E}}_{S}$$ (15)

$$\sigma_{1}{\tilde{F}}_{S}⊕\sigma_{2}{\tilde{F}}_{S}=(\sigma_{1}+\sigma_{2}){\tilde{F}}_{S}$$ (16)

$$({{\tilde{F}}_{S}⊗{\tilde{E}}_{S})}^{\sigma }={\tilde{F}}_{S}^{\sigma }⊗{\tilde{E}}_{S}^{\sigma }$$ (17)

$${\tilde{F}}_{S}^{\sigma_{1}}⨂{\tilde{F}}_{S}^{\sigma_{2}}={\tilde{F}}_{S}^{\sigma_{1}+\sigma_{2}}$$ (18)

Definition 4. Spherical weighted arithmetic mean (SWAM) concerning $w=(w_{1},w_{2},I,w_{n});\;w_{i}\in [0,1]$; $\sum_{i=1}^{n}w_{i}=1$, SWAM is defined in Eq (19):

$${SWAM}_{w}({\tilde{F}}_{S1},\ldots ,{\tilde{F}}_{Sn})=w_{1}{\tilde{F}}_{S1}+w_{2}{\tilde{F}}_{S2}+I+w_{n}{\tilde{F}}_{Sn}=\left\{\sqrt{\left[1-{\prod_{i=1}^{n}(1-\alpha_{{\tilde{F}}_{Si}}^{2})}^{w_{i}}\right]},\prod_{i=1}^{n}\beta_{{\tilde{F}}_{Si}}^{w_{i}},\sqrt{\left[{\prod_{i=1}^{n}(1-\alpha_{{\tilde{F}}_{Si}}^{2})}^{w_{i}}-{\prod_{i=1}^{n}(1-\alpha_{{\tilde{F}}_{Si}}^{2}-\gamma_{{\tilde{F}}_{Si}}^{2})}^{w_{i}}\right]}\right\}$$ (19)

Definition 5. Spherical weighted geometric mean (SWGM) concerning $w=(w_{1},w_{2}I,w_{n};\;w_{i}\in [0,1];\;\sum_{i=1}^{n}w_{i}=1$, SWGM is defined in Eq (20):

$${SWGM}_{w}{(\tilde{F}}_{S1},\ldots ,{\ddot{F}}_{Sn})={\tilde{F}}_{S1}^{w_{1}}+{\tilde{F}}_{S2}^{w_{2}}+I+{\tilde{F}}_{Sn}^{w_{n}}=\left\{\prod_{i=1}^{n}\alpha_{{\tilde{F}}_{Si}}^{w_{i}},\sqrt{\left[1-{\prod_{i=1}^{n}(1-\beta_{{\tilde{F}}_{Si}}^{2})}^{w_{i}}\right]},\sqrt{\left[{\prod_{i=1}^{n}(1-\beta_{{\tilde{F}}_{Si}}^{2})}^{w_{i}}-{\prod_{i=1}^{n}(1-\beta_{{\tilde{F}}_{Si}}^{2}-\gamma_{{\tilde{F}}_{Si}}^{2})}^{w_{i}}\right]}\right\}$$ (20)

### 3.3. Proposed method

**Fig 2:**
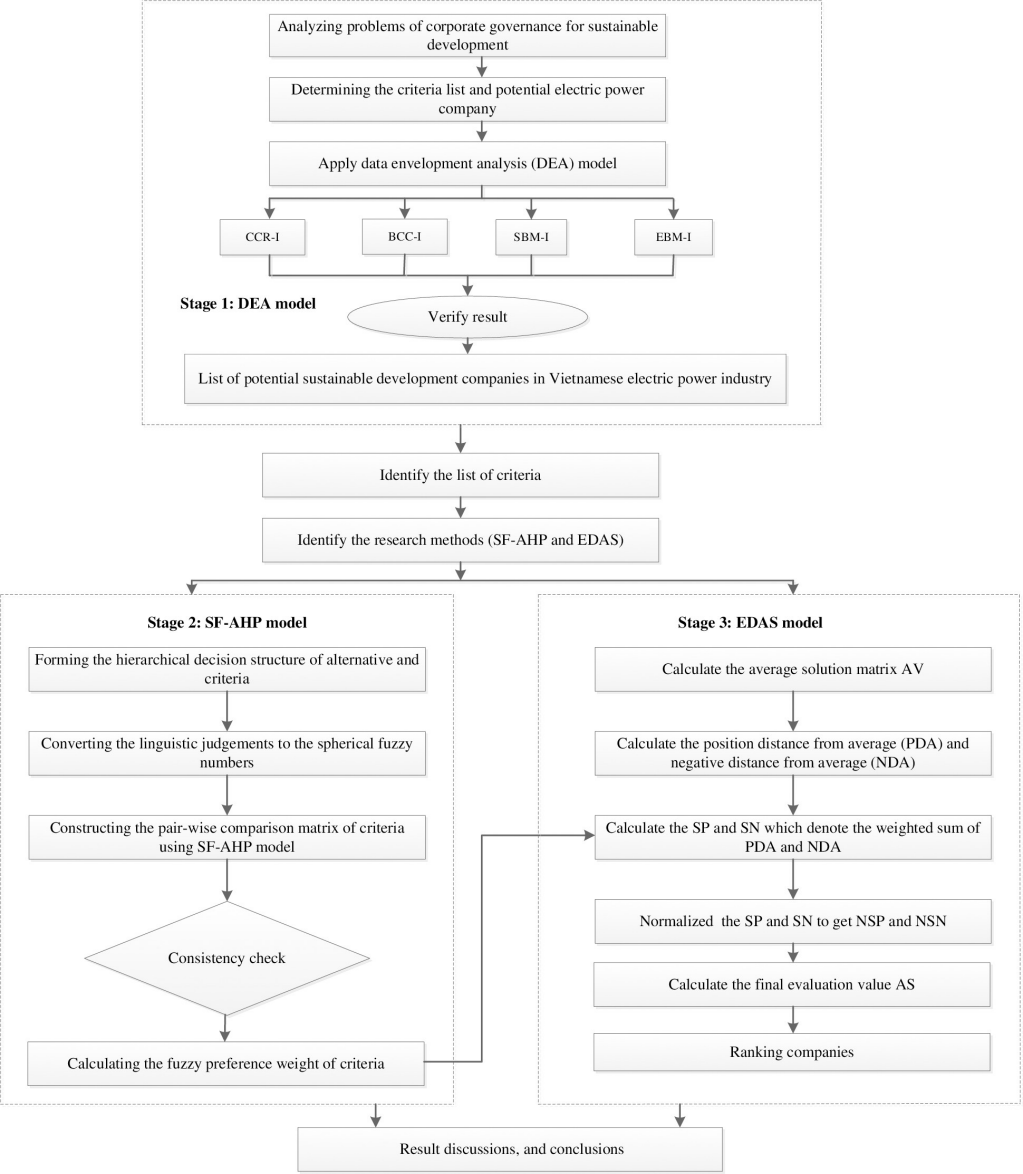
Research framework.

The study framework is structured into three distinct stages, as depicted in Fig 2.

Stage 1: Screening potential enterprises with DEA models.

The DEA models, namely CCR, BCC, SBM, and EBM, discussed in Section 3.1, are utilized to identify potential enterprises from a list of 15 electric power enterprises in Vietnam by analyzing their financial indicators. This study considers three inputs (total asset, equity, number of employees) and one output (revenue) based on expert interviewing and literature reviews. The definitions of input and output factors are explained as follows:

(I1) Total Assets: The total assets owned by electric power enterprises [55].

(I2) Equity: The quantity of capital that a company’s owner has invested or possesses [55].

(I3) Number of employees: The total number of employees, both full-time and part-time, present at a business location on each normal working day for a given calendar year [64].

(O1) Revenue: The regular earnings generated by the company [55].

Stage 2: SF-AHP model

The sustainable growth of specific electric power enterprises is analyzed using the SF-AHP model, which incorporates expert judgment to handle uncertainties and ambiguity. The analysis focuses on aspects such as Business Management, Corporate Social Responsibility, and Corporate Governance Shareholder. In order to verify the validation of the model, the coherence of the pairwise comparison matrices is evaluated. The SF-AHP methodology is explicated as follows:

Step 1: The hierarchical framework is structured such that the study goal is situated at level 1, while the recommended criteria are positioned accordingly *C* = {*C*_1_,*C*_2_,I*C*_*n*_} with *n*≥2 in level 2.

Step 2: The process of conducting pairwise comparison matrices involves the use of linguistic scales, as seen in Table 2. The calculation of score indices (SI) is performed using Eqs (21) and (22):

$$SI=\sqrt{\left|100*[{\left(\alpha_{{\tilde{F}}_{S}}-\gamma_{{\tilde{F}}_{S}}\right)}^{2}-({\beta_{{\tilde{F}}_{S}}-\gamma_{{\tilde{F}}_{S}})}^{2}]\right|}$$ (21)

for AMI, VHI, HI, SMI, and EI.

$$\frac{1}{SI}=\frac{1}{\sqrt{\left|100*[{\left(\alpha_{{\tilde{F}}_{S}}-\gamma_{{\tilde{F}}_{S}}\right)}^{2}-({\beta_{{\tilde{F}}_{S}}-\gamma_{{\tilde{F}}_{S}})}^{2}]\right|}}$$ (22)

for EI, SLI, LI, VLI, and ALI.

**Table 2:** SF-AHP linguistic terms.

Scales	(*α*,*β*,*γ*)	Score Index (SI)
Absolutely More Importance (AMI)	(0.9, 0.1, 0.0)	9
Very High Importance (VHI)	(0.8, 0.2, 0.1)	7
High Importance (HI)	(0.7, 0.3, 0.2)	5
Slightly More Importance (SMI)	(0.6, 0.4, 0.3)	3
Equally Importance (EI)	(0.5, 0.4, 0.4)	1
Slightly Low Importance (SLI)	(0.4, 0.6, 0.3)	1/3
Low Importance (LI)	(0.3, 0.7, 0.2)	1/5
Very Low Importance (VLI)	(0.2, 0.8, 0.1)	1/7
Absolutely Low Importance (ALI)	(0.1, 0.9, 0.0)	1/9

Step 3: To maintain the validity of pairwise comparison matrices, it is necessary to conduct consistency tests to verify that the consistent ratio (CR) remains below 10% when compared to the random index (RI) as outlined in Table 3.

**Table 3:** RI values.

N	1	2	3	4	5	6	7	8	9	10	11	12	13	14	15
RI	0	0	0.58	0.9	1.12	1.24	1.32	1.41	1.45	1.49	1.51	1.48	1.56	1.57	1.59

Where, n is the number of criteria, RI signifies the random index.

Step 4: The weight of each factor/criterion can be determined using the SWAM operator, as specified by Eq (19).

Step 5: The crisp weights of the final criteria rankings are determined using Eq (23). The criteria weights can be normalized using Eq (24) and subsequently applied to the spherical fuzzy multiplication as described in Eq (25).


$$S({\tilde{w}}_{j}^{s})=\sqrt{\left|100*[{\left(3\alpha_{{\tilde{F}}_{S}}-\frac{\gamma_{{\tilde{F}}_{S}}}{2}\right)}^{2}-{\left(\frac{\beta_{{\tilde{F}}_{S}}}{2}-\gamma_{{\tilde{F}}_{S}}\right)}^{2}]\right|}$$ (23)

$${\bar{w}}_{j}^{s}=\frac{S({\tilde{w}}_{j}^{s})}{\sum_{j=1}^{n}S({\tilde{w}}_{j}^{s})}$$ (24)

$${\tilde{F}}_{S_{ij}}={\bar{w}}_{j}^{s}.{\tilde{F}}_{S_{i}}=\left\{\sqrt{{\left(1-(1-\alpha_{{\tilde{F}}_{S}}^{2}\right)}^{w_{j}^{-s}})},\beta_{{\tilde{F}}_{S}}^{{\bar{w}}_{j}^{s}},\sqrt{{\left(1-\alpha_{{\tilde{F}}_{S}}^{2}\right)}^{w_{j}^{-s}}-{\left(1-\alpha_{{\tilde{F}}_{S}}^{2}-\gamma_{{\tilde{F}}_{S}}^{2}\right)}^{w_{j}^{-s}})}\right\}\forall i$$ (25)

Stage 3: Ranking via the EDAS method

The EDAS method was formulated by Keshavarz Ghorabaee et al. in 2015 [56]. The method is highly valuable when there are conflicting criteria. According to the EDAS technique, the optimal alternative is determined based on its deviation from the average solution (AV). Two measures must be calculated to assess the desirability of the alternatives: the positive distance from average (PDA) and the negative distance from average (NDA). The steps of this method are stated as follows, where n represents the number of alternatives and m represents the number of criteria.

Step 1. Create the decision-making matrix

$$=[X_{ij}]=\left[\begin{array}{cccc} X_{11} & X_{12} & \cdots & X_{1n}\\ X_{21} & X_{22} & \cdots & X_{2m}\\ \vdots & \vdots & \ddots & \vdots \\ X_{n1} & X_{n2} & \cdots & X_{nm} \end{array}\right]$$ (26)

Where, the performance value of the *ith* alternative on the *jth* criterion is denoted as*X*_*ij*_.

Step 2: Calculate the mean answer based on all criteria, as indicated below:

$$AV={\left[AV_{j}\right]}_{1xm}$$ (27)

$$where,\;{AV}_{j}=\frac{\sum_{i=1}^{n}X_{ij}}{n}$$ (28)

Step 3. Compute the PDA and NDA matrices based on the criteria type (benefit and cost), as illustrated below:

$$PDA={\left[{PDA}_{ij}\right]}_{nxm};$$ (29)

$$NDA={\left[{NDA}_{ij}\right]}_{nxm};$$ (30)

If *jth* criterion is beneficial,

$${PDA}_{ij}=\frac{max(0,(X_{ij}-{AV}_{j}))}{{AV}_{j}};$$ (31)

$${NDA}_{ij}=\frac{max(0,({AV}_{ij}-X_{j}))}{{AV}_{j}}$$ (32)

If *j*_*th*_ criterion is cost (non-beneficial),

$${PDA}_{ij}=\frac{max(0,({AV}_{ij}-X_{j}))}{{AV}_{j}};$$ (33)

$${NDA}_{ij}=\frac{max(0,(X_{ij}-{AV}_{j}))}{{AV}_{j}}$$ (34)

where *PDA*_*ij*_ and *NDA*_*ij*_ represent the positive and negative distances, respectively, of the ith alternative from the average solution in terms of the *jth* criterion.

Step 4. Calculate the weighted sum of PDA and NDA for each alternative.

$${SP}_{i}=\sum_{j=1}^{m}w_{j}\;{PDA}_{ij};$$ (35)

$${SN}_{i}=\sum_{j=1}^{m}w_{j}\;{NDA}_{ij};$$ (36)

where *w*_*j*_ represents the relative weight of the *jth* criterion.

Step 5. Normalize the variables of SP and SN for all options, as demonstrated below:

$${NSP}_{i}=\frac{{SP}_{i}}{{max}_{i}({SP}_{i})};$$ (37)

$${NSN}_{i}=1-\frac{{SN}_{i}}{{max}_{i}({SN}_{i})};$$ (38)

Step 6. Determine the appraisal score (AS) for each alternative, as presented below:

$${AS}_{i}=\frac{{NSP}_{i}+{NSN}_{i}}{2}$$ (39)

Where 0≤*AS*_*i*_≤1.

Step 7. Arrange the choices in descending order based on the values of AS. The candidate alternative with the highest AS (Alternative Score) is the optimal decision.

## 4. Case study

Electricity is essential to all aspects of contemporary human existence. Consequently, it has a significant impact on the progress of entire national economies, particularly in the context of industrialization. At the national level, electricity plays a crucial role in enhancing the standard of living and fostering equal opportunities, hence reducing the disparity between urban and rural populations. Thus, ensuring that everyone has access to energy is frequently seen as a crucial factor in promoting inclusive economic development.

Electricity of Vietnam (EVN) reported that the total power generation for the entire system in July 2023 amounted to 26.20 billion kWh, representing a 7.1% growth compared to the corresponding period in 2022. During the initial 7 months of 2023, the total power generation of the entire system amounted to 160.58 billion kWh, representing a growth of 1.9% compared to the corresponding period in 2022 [65]. Based on the report from VIRAC and data supplied by EVN, the cumulative power generation from coal fuel during the initial four months of 2023 amounted to 40.0 billion kWh, representing 46.5% of the overall electricity production and import of the entire system. In addition, as per VIRAC’s study, coal-fired power generation had a 1% decline in the first quarter of 2023 compared to the comparable time in the previous year. In the domestic market, despite fully mobilizing the supply of fuel to improve power output, the demand for electricity still exceeds the available supply, resulting in an inability to meet operational needs. The power development plan aims to meet the electricity needs of the country and contribute to its socio-economic development objectives, with an average annual GDP growth rate of around 7% between 2021 and 2030, and a range of 6.5–7.5% between 2031 and 2050 [66]. Sustainable development seeks to enhance the well-being of individuals while ensuring the preservation of natural resources by avoiding excessive exploitation. The concept involves implementing measures and modifying regulations and procedures, spanning from the personal to the global scale. Sustainable development has emerged as a prominent focus of political agendas globally, with nearly all governments dedicated to merging economic well-being, environmental preservation, and social harmony [67]. This study aims to validate the indicators of corporate governance for sustainable development (CGS) for the Vietnamese electric power firm. It will examine 21 criteria by comparing them with those of 10 Vietnamese electric power enterprises in terms of sustainable development. The selection of CGS indicators will be determined by 12 expert’s discretion and guided by the literature. The proposed indicators will undertake validation by expert questionnaires in this field.

Instead of employing the conventional method of evaluating productivity efficiency, consider a different technique where the inputs are elements that are anticipated to enhance as their values decline, and the outputs are those that are expected to improve when their values increase [68]. Then, the dataset comprising the input and output features of 15 enterprises has been gathered from the OECD ilibrary [69]. The unit of measurement used in the dataset is VND 1 million, as depicted in Table 4.

**Table 4:** The dataset of 15 companies for the Vietnamese electric power industry.

Company	(I)Total asset	(I)Equity	(I)Number of employees	(O)Total revenue
Vietnam Electricity	510,338,000	216,684,000	4974	332,030,000
Power Generation Corporation 1	97,739,000	26,091,000	3280	39,769,000
Power Generation Joint Stock Corporation 2	51,045,000	22,561,000	3029	26,348,000
Power Generation Joint Stock Corporation 3	72,900,000	14,964,000	2778	40,367,000
Thu Duc Electro Mechanical JSC	366,000	122,000	136	111,000
National Power Transmission Corporation	85,298,000	25,220,000	7114	18,021,000
Northern Power Corporation Contact Center	77,096,000	22,345,000	26416	131,092,000
Central Power Corporation	34,150,000	10,785,000	11432	36,484,000
Southern Power Corporation	41,828,000	17,529,000	21710	134,644,000
Hanoi City Power Corporation	32,484,000	10,701,000	7459	41,126,000
Hochiminh City Power Corporation	26,628,000	12,809,000	6585	54,392,000
Power Engineering Consulting JSC 1	1,602,000	280,000	674	632,000
Power Engineering Consulting JSC 2	3,336,000	1,167,000	963	3,346,000
Power Engineering Consulting JSC 3	319,000	116,000	469	404,000
Power Engineering Consulting JSC 4	336,000	186,000	426	251,000
Dong Anh Electrical Equipment Corporation	1,443,000	608,000	748	2,422,000

**Fig 3:**
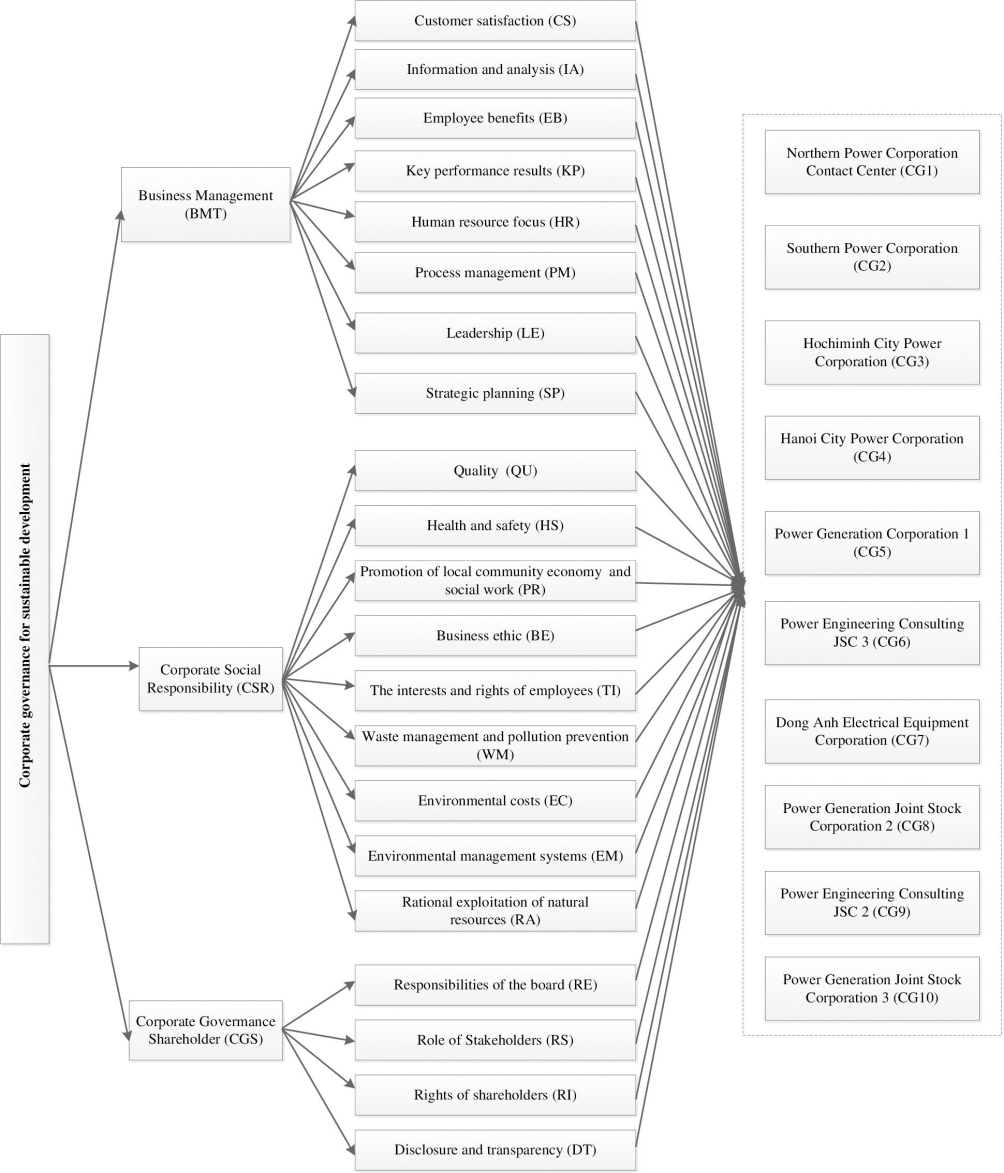
The main and sub-criteria hierarchy.

During the second phase of the suggested approach, the main criteria and sub-criteria are established to assess the efficacy of sustainable business development management serving state-owned electricity enterprises in Vietnam. A hierarchical structure consisting of two levels is formed by taking into account the criteria and their corresponding sub-criteria. A detailed analysis is conducted by considering a total of twenty-one distinct sub-criteria. The aforementioned criteria are established by a comprehensive examination of relevant literature and by seeking input from a panel of experts in the Vietnamese electric power industry. The study incorporates the criteria outlined in Fig 3, which is presented in a hierarchical framework.

In order to conduct a thorough assessment, the study attempts to incorporate all factors that may influence the decision-making process. In order to address this problem, a total of twenty-one different variables have been identified as sub-criteria. These aspects are further divided into three main criteria, namely business management, corporate social responsibility, and corporate governance shareholder. Table 5 defines three main criteria and twenty-one sub-criteria.

**Table 5:** Criteria and sub-criteria descriptions.

Criteria	Sub-Criteria	Definition	References
Business Management (BMT)	Customer satisfaction (CS)	A metric that signifies the degree of customer satisfaction regarding the offerings, services, and competencies of an organization.	[70]
	Information and analysis (IA)	The process of gathering, organisation, examining, and evaluating data or information in order to obtain valuable insights and make informed and accurate decisions.	[71]
	Employee benefits (EB)	Any form of remuneration, whether tangible or intangible, provided to personnel in addition to their basic wages or salaries.	[72]
	Key Performance Results (KP)	The outcome you should expect to see as a result of the activities (KPIs) that are being conducted on a regular basis.	[71]
	Human resource focus (HR)	The process of personnel recruitment and maintaining a workforce within an organization.	[71]
	Process management (PM)	Aligning processes with strategic goals, creating and executing process architectures, building process measurement tools, and teaching and organisation managers to manage processes effectively.	[71]
	Leadership (LE)	The ability to influence and lead followers or members of an organisation, society, or team.	[71]
	Strategic planning (SP)	The procedure by which the executives of an organisation establish their vision for the future and determine the goals and objectives of the organization.	[71]
Corporate social responsibility (CSR)	Quality (QU)	Adherence to the prescribed standards. The extent to which a product satisfies all customer expectations and meets the design requirements, thereby generating a fulfilment factor.	[70]
	Health and safety (HS)	It prioritises the health, safety, and overall welfare of employees within the organization’s physical space.	[55]
	Promotion of local community economy and social work (PR)	Assisting in alleviating human distress, advocating for social justice, and enhancing communities and lives through initiatives such as child welfare and poverty reduction, etc.	[73]
	Business ethic (BE)	The enforcement of procedures and rules pertaining to subjects including corporate governance, bribery, fraud, and discrimination.	[72]
	The interests and rights of employees (TI)	The focus on various aspects and prerequisites pertaining to employees that contribute to the long-term sustainability and performance of an organization.	[55]
	Waste management and pollution prevention (WM)	The selection of the primary material is organisation to reduce pollution and waste throughout the manufacturing process.	[55]
	Environmental costs (EC)	The effort to minimize costs and mitigate environmental impact throughout the whole lifecycle of both raw materials and final products.	[55]
	Environmental management systems (EM)	The framework, technologies, and execution of organisation environmental protection strategies.	[55]
	Rational exploitation of natural resources (RA)	Exploiting and preserving natural resources to assure their survival and future use, increase economic efficiency, community benefits, and environmental sustainability.	[74]
Corporate Govermance Shareholder (CGS)	Responsibilities of the Board (RE)	The responsibility of making comprehensive policy decisions and offering supervision lies with the shareholder.	[55]
	Role of Stakeholders (RS)	Assisting an organisation in achieving its strategic goals through the provision of expertise and insights to a specific project.	[75]
	Rights of Shareholders (RI)	The right to view the company’s books and records, sue for executives and officers’ wrongdoing, and vote on important corporate affairs including board director appointments.	[75]
	Disclosure and Transparency (DT)	Encourage transparency by delivering regular updates to shareholders and fellow investors; these reports serve as a significant source of information for investors.	[70]

## 5. Results and discussions

### 5.1. Results

#### 5.1.1. DEA models’ results

The gathered data will be employed to validate the CCR-I, BCC-I, SBM-I-C, and EBM-I models. The purpose of this stage is to identify the state-owned electricity firms in Vietnam ((DMUs) that have the highest average score in sustainable business development management, using the most critical financial indicators and number of employees indicator listed in Table 4. The efficiency scores of DMUs running in DEA modes are presented in Table 6.

**Table 6:** Enterprise efficiency ratings in the DEA models.

DMUs	BBC-I	CCR-I	SBM-I-C	EBM-I-C	Average	Ranking
Power Generation Corporation 1	0.835	0.834	0.702	0.784	0.7887995	4
Power Generation Joint Stock Corporation 2	0.743	0.732	0.542	0.685	0.6755295	7
Power Generation Joint Stock Corporation 3	1.000	1.000	1.000	1.000	1	1
Thu Duc Electro Mechanical JSC	1.000	0.128	0.115	0.124	0.341822	13
National Power Transmission Corporation	0.249	0.241	0.189	0.238	0.229224	15
Northern Power Corporation Contact Center	0.795	0.791	0.697	0.757	0.7600435	5
Central Power Corporation	0.502	0.495	0.429	0.476	0.475497	11
Southern Power Corporation	1.000	1.000	1.000	1.000	1	1
Hanoi City Power Corporation	0.757	0.751	0.594	0.721	0.70599075	6
Hochiminh City Power Corporation	1.000	1.000	1.000	1.000	1	1
Power Engineering Consulting JSC 1	0.582	0.294	0.189	0.251	0.328961	14
Power Engineering Consulting JSC 2	0.609	0.501	0.415	0.485	0.50268825	10
Power Engineering Consulting JSC 3	1.000	0.453	0.329	0.403	0.5462235	9
Power Engineering Consulting JSC 4	0.972	0.232	0.168	0.206	0.3945105	12
Dong Anh Electrical Equipment Corporation	0.733	0.522	0.521	0.522	0.5743505	8

The results of the DEA analysis regarding the efficiency scores of the DMUs are summarized in Table 6. Thus, the DEA analysis provides perfect average efficiency scores to a collective of ten DMUs: Power Generation Corporation 1, Power Generation Joint Stock Corporation 2, Power Generation Joint Stock Corporation 3, Northern Power Corporation Contact Center, Southern Power Corporation, Hanoi City Power Corporation, Hochiminh City Power Corporation, Power Engineering Consulting JSC 2, Power Engineering Consulting JSC 3, and Dong Anh Electrical Equipment Corporation. In light of the fact that these ten DMUs are regarded as the most promising for sustainable business development in the electric power sector of Vietnam, have been selected for evaluation in the next phase using SF-AHP and EDAS models.

#### 5.1.2. SF-AHP and EDAS model’s results

To establish the priority ranking of the 10 different Vietnamese electric power industry options, the criteria were initially assigned weights using the SF-AHP approach. To address this objective, an expert decision-making team specializing in the management of corporate sustainable development in state-owned electric power enterprises was established. Their role is to provide answers to the pairwise comparisons matrix. This study presents a hierarchical structure comprising of 21 sub-criteria categorized under three primary criteria for the purpose of prioritizing firms. Due to the long nature of the SF-AHP approach, the study provides a detailed explanation of the procedure phases based on the main criteria. The consistency ratios (CR) of the pairwise comparison matrices are calculated using the SF-AHP technique. Preliminary pairwise comparisons were performed in Table 7 using the questionnaire responses.

**Table 7:** Initial comparison matrix of main criteria.

	Left Criteria Are Important					Right Criteria Are Important					Experts
	9	7	5	3	1	1/3	1/5	1/7	1/9		
Criteria	AMI	VHI	HI	SMI	EI	SLI	LI	VLI	ALI	Criteria	
BMT		5	2	4	1					CSR	12
CSR	3	4	2	3						CGS	12
CGS		3	2	1	6					CGS	12

Tables 8 and 9 display the Crisp matrix and Normalized matrix for CR, respectively.

**Table 8:** Crisp matrix.

Criteria	BMT	CSR	CGS
BMT	1.000	4.243	5.702
CSR	0.236	1.000	2.331
CGS	0.175	0.429	1.000

**Table 9:** Normalized matrix.

	BMT	CSR	CGS	MEAN	WSV	CV
BMT	0.709	0.748	0.631	0.6960	2.1369	3.0703
CSR	0.167	0.176	0.258	0.2005	0.6059	3.0223
CGS	0.124	0.076	0.111	0.1035	0.3116	3.0094

Then,

$$\lambda_{max}=\frac{3.0703+3.0223+3.0094}{3}=3.0340$$

$$CI=\frac{\lambda_{max}-n}{n-1}=\frac{3.0340-3}{3-1}=0.0170$$

With n = 3 and RI = 0.58, CR value is computed as

$$CR=\frac{CI}{RI}=\frac{0.0170}{0.58}=0.0293$$

With CR = 0.0293 < 0.1, the outcome is acceptable

Then, Table 10 depicts the weights assigned to the main criteria

**Table 10:** The weights of main criteria.

	Spherical Fuzzy Weights			Crisp Weights	Rank
BMT	0.664	0.324	0.277	0.450	1
CSR	0.491	0.462	0.331	0.317	2
CGS	0.371	0.590	0.312	0.233	3

The local weights of each sub-criterion are determined for all sub-criteria. Following these computations, the major criteria weights are multiplied by the sub-criteria weights, resulting in the determination of the global weights for all sub-criteria. The weights for both local and global variables are provided in Table 11.

**Table 11:** Results of the final rankings and weights.

Criteria	SF-Wc	M-w	Rank	Sub-Criteria	SF-Ws	L-w	Rank	G-w	G-crisp-W	Rank
BMT	(0.664,0.324,0.277)	0.450	1	CS	(0.420, 0.570, 0.288)	0.105	7	0.047	0.051	10
				IA	(0.416, 0.564, 0.305)	0.103	8	0.046	0.051	11
				EB	(0.440, 0.549, 0.295)	0.110	5	0.050	0.053	8
				KP	(0.430, 0.550, 0.307)	0.107	6	0.048	0.052	9
				HR	(0.485, 0.476, 0.330)	0.121	4	0.054	0.059	6
				PM	(0.517, 0.462, 0.315)	0.131	3	0.059	0.063	5
				LE	(0.602, 0.381, 0.295)	0.156	2	0.070	0.073	3
				SP	(0.639, 0.350, 0.271)	0.167	1	0.075	0.077	2
CSR	(0.491, 0.462, 0.331)	0.317	2	QU	(0.513, 0.463, 0.320)	0.120	1	0.038	0.046	13
				HS	(0.501, 0.484, 0.313)	0.118	3	0.037	0.045	15
				PR	(0.496, 0.485, 0.322)	0.115	5	0.037	0.044	17
				BE	(0.506, 0.471, 0.326)	0.119	2	0.038	0.045	14
				TI	(0.499, 0.471, 0.326)	0.116	4	0.037	0.045	16
				WM	(0.437, 0.536, 0.371)	0.101	8	0.032	0.039	20
				EC	(0.462, 0.520, 0.306)	0.107	6	0.034	0.041	18
				EM	(0.452, 0.520, 0.323)	0.104	7	0.033	0.041	19
				RA	(0.433, 0.541, 0.311)	0.100	9	0.032	0.039	21
CGS	(0.371, 0.590, 0.312)	0.233	3	RE	(0.645, 0.338, 0.290)	0.329	1	0.076	0.044	1
				RS	(0.447, 0.523, 0.317)	0.218	3	0.051	0.030	7
				RI	(0.555, 0.417, 0.315)	0.277	2	0.064	0.038	4
				DT	(0.368, 0.609, 0.290)	0.176	4	0.041	0.025	12

Firstly, the consistency of the pairwise comparison matrix is assessed. To achieve this objective, it is established that the matrix is consistent. Next, the SF-AHP method is utilized to calculate the weights of the main criterion. The weights assigned to the main criteria of Business Management, Corporate Social Responsibility, and Corporate Governance Shareholder are 0.450, 0.317, and 0.233, respectively. The main criteria with the greatest significance are identified as business management, with a weight of 0.045. Corporate social responsibility is also an importance factor, carrying a weight of 0.317. The corporate governance shareholder criterion holds the lowest level of significance, with a weight of 0.233, compared to other main criteria.

When Table 11 is examined further, based on the local weight ranking, a scale measure showing the aspect of business management for which strategic planning is considered to have the highest weight according to expert opinion. Furthermore, the corporate social responsibility aspect considers quality as the most important factor. The responsibility of the board is the most important task of the corperate governance shareholder aspect.

Ultimately, the final global weights reveal the significance of one criterion in comparison to the other criteria. The relative importance of the criteria is displayed in Table 11. The primary metric of significance relates to the responsibilities of the board in electric power enterprises. The second indicator represents strategic planning, while the third indicator signifies leadership among electric power enterprises, followed by rights of shareholders and process management factors. Based on the findings provided by the experts, it is evident that the criteria related to the rational exploitation of natural resources carries the least amount of significance.

Then, ten alternatives are assessed utilizing the EDAS procedure that complies with the established criteria. By employing this approach, we initially derive the average solution based on all criteria by utilizing Eq (28). The outcomes of this stage are documented and illustrated in Table 12 referring to each criterion. Eqs (29)–(30) are utilized in the second phase to determine the positive and negative distances from the mean for each alternative in relation to the benefit and cost criteria, respectively. The outcomes pertaining to the positive and negative deviations from the mean are presented in Tables 13 and 14, respectively. As shown in Tables 13, 14, we calculate weighted PDA and NDA for each of the alternatives denoted as Spi and Sni using Eqs (35)–(36). The Spi and Sni values are then normalized and presented in Table 15 as NSNi and NSPi, respectively, using Eqs (37)–(38). The final stage involves the computation of the appraisal score for each alternative utilizing the equation Eqs (39), which denotes their ranking.

**Table 12:** The average answer based on all the factors.

	CS	IA	EB	KP	HR	PM	LE	SP	QU	HS	PR	BE	TI	WM	EC	EM	RA	RE	RS	RI	DT
Avj	2.05	2.44	3.24	2.50	2.67	2.79	1.78	1.75	3.03	2.98	1.90	2.57	2.87	3.13	2.96	2.78	2.42	2.77	2.46	2.46	2.28

**Table 13:** Positive Distance from Average (PDA).

ALT	CS	IA	EB	KP	HR	PM	LE	SP	QU	HS	PR	BE	TI	WM	EC	EM	RA	RE	RS	RI	DT
CG1	0.131	0.636	0.000	0.202	0.000	0.794	0.061	0.718	0.431	0.006	0.000	0.429	0.000	0.000	0.000	0.080	0.000	1.047	0.000	0.220	1.192
CG2	0.245	0.000	0.424	0.000	0.125	0.555	0.000	0.000	0.000	0.454	0.000	0.169	0.000	0.596	0.000	1.279	0.000	0.325	0.000	1.033	0.000
CG3	0.136	0.000	0.544	0.000	0.625	0.000	0.000	0.000	0.000	0.006	0.000	0.000	0.047	0.000	0.000	0.799	0.000	0.000	0.220	1.033	1.280
CG4	0.000	0.227	0.000	0.000	0.750	0.000	0.684	0.527	0.000	0.000	0.581	0.000	0.512	0.702	0.000	0.000	1.621	0.000	0.762	0.220	0.000
CG5	0.786	0.000	0.000	1.004	1.125	0.000	0.310	0.000	0.000	0.677	0.000	0.000	0.453	0.000	0.000	0.000	0.000	0.084	0.762	0.000	0.000
CG6	0.000	0.227	0.544	0.000	0.000	0.000	0.000	0.000	0.000	0.000	0.581	0.000	0.047	0.000	0.700	0.000	0.517	0.000	0.000	0.220	0.000
CG7	0.239	1.045	0.029	1.271	0.000	0.794	0.000	0.000	0.431	0.000	0.000	0.039	0.000	0.596	0.000	0.000	0.379	0.000	0.220	0.000	0.315
CG8	0.000	0.000	0.000	0.000	0.000	0.000	0.248	0.000	0.000	0.006	0.171	0.169	0.279	0.000	0.726	0.559	0.241	0.084	1.033	0.000	0.000
CG9	0.055	0.227	0.029	0.000	0.000	0.316	0.684	0.718	0.000	0.006	0.000	0.078	0.570	0.000	0.663	0.000	0.000	0.084	0.000	0.000	0.000
CG10	0.000	0.000	0.000	0.202	0.000	0.077	0.000	0.718	0.000	0.230	0.581	0.689	0.047	0.000	0.685	0.799	0.241	0.084	0.000	0.000	0.315

**Table 14:** Negative Distance from Average (NDA).

	CS	IA	EB	KP	HR	PM	LE	SP	QU	HS	PR	BE	TI	WM	EC	EM	RA	RE	RS	RI	DT
CG1	0.000	0.000	0.485	0.000	0.625	0.000	0.000	0.000	0.000	0.000	0.122	0.000	0.651	0.043	0.125	0.000	0.586	0.000	0.593	0.000	0.000
CG2	0.000	0.591	0.000	0.599	0.000	0.000	0.501	0.427	0.009	0.000	0.122	0.000	0.651	0.000	0.012	0.000	0.609	0.000	0.621	0.000	0.562
CG3	0.000	0.364	0.000	0.461	0.000	0.641	0.195	0.459	0.229	0.000	0.473	0.406	0.000	0.255	1.362	0.000	0.586	0.157	0.000	0.000	0.000
CG4	0.351	0.000	0.177	0.599	0.000	0.103	0.000	0.000	0.064	0.665	0.000	0.372	0.000	0.000	1.137	0.640	0.000	0.687	0.000	0.000	0.591
CG5	0.000	0.591	0.125	0.000	0.000	0.402	0.000	0.277	0.009	0.000	0.602	0.143	0.000	0.043	0.012	0.728	0.632	0.000	0.000	0.638	0.620
CG6	0.540	0.000	0.000	0.087	0.625	0.749	0.414	0.459	0.009	0.236	0.000	0.654	0.000	0.716	0.000	0.764	0.000	0.177	0.593	0.000	0.391
CG7	0.000	0.000	0.000	0.000	0.625	0.000	0.439	0.567	0.000	0.486	0.122	0.000	0.651	0.000	0.125	0.664	0.000	0.687	0.000	0.616	0.000
CG8	0.188	0.227	0.708	0.599	0.125	0.641	0.000	0.491	0.450	0.000	0.000	0.000	0.000	0.681	0.000	0.000	0.000	0.000	0.000	0.096	0.591
CG9	0.000	0.000	0.000	0.332	0.000	0.000	0.000	0.000	0.083	0.000	0.473	0.000	0.000	0.043	0.000	0.720	0.586	0.000	0.593	0.661	0.347
CG10	0.513	0.591	0.074	0.000	0.625	0.000	0.439	0.000	0.009	0.000	0.000	0.000	0.000	0.113	0.000	0.000	0.000	0.000	0.593	0.715	0.000

**Table 15:** EDAS computations result.

	Spi	Sni	NSPi	NSNi	Asi	Rank
CG1	1.062	0.424	1.000	0.526	0.763	1
CG2	0.820	0.661	0.772	0.261	0.517	2
CG3	0.799	0.690	0.753	0.229	0.491	3
CG4	0.894	0.783	0.842	0.125	0.484	4
CG5	0.700	0.692	0.659	0.227	0.443	5
CG6	0.344	0.895	0.324	0.000	0.162	10
CG7	0.633	0.865	0.596	0.034	0.315	9
CG8	0.524	0.620	0.494	0.308	0.401	7
CG9	0.475	0.612	0.448	0.317	0.382	8
CCG10	0.597	0.607	0.563	0.322	0.442	6

**Fig 4:**
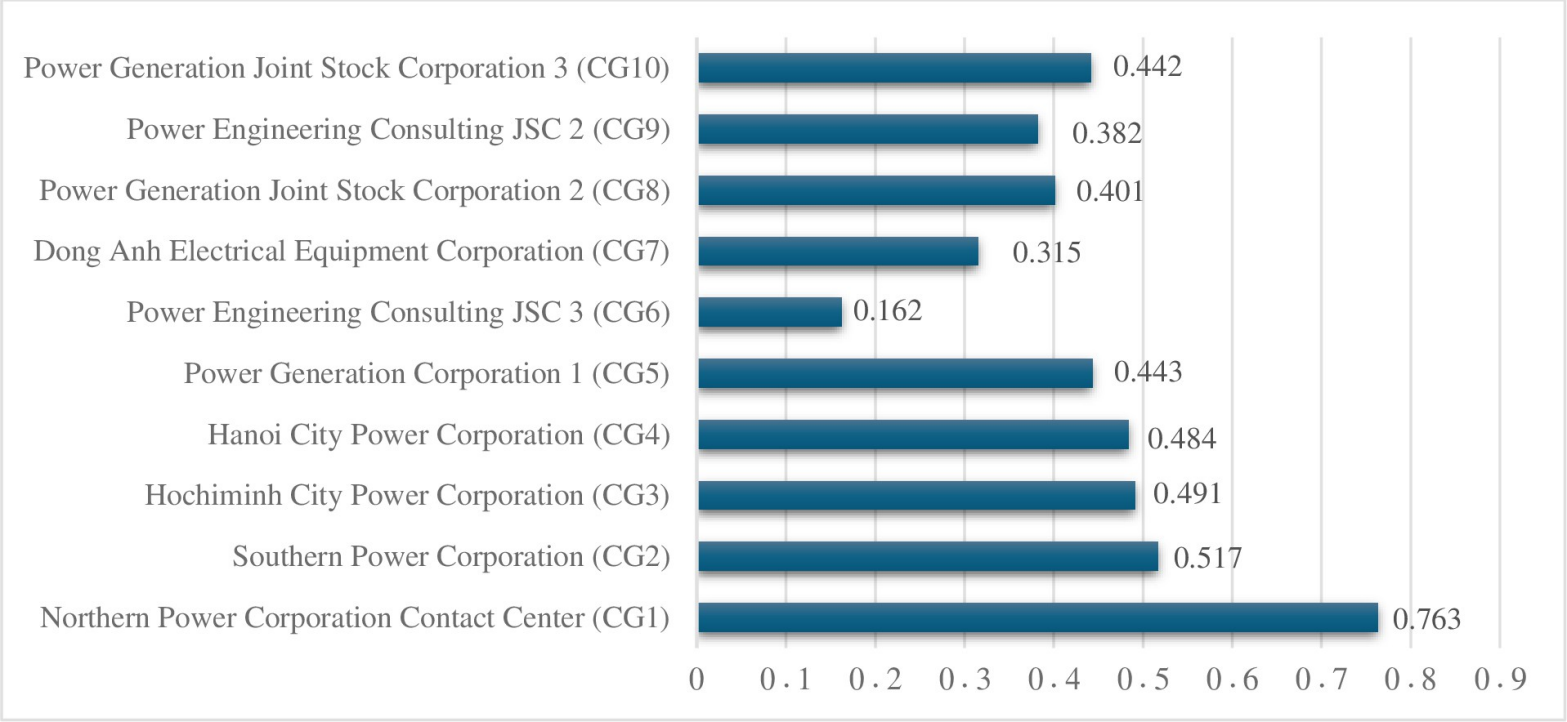
Appraisal score (AS).

The findings derived from the suggested integrated decision-making approach indicate that Northern Power Corporation Contact Center (CG1) emerges as the most promising and best-performing solution among the many options, as evidenced by its highest appraisal score. The Southern Power Corporation (CG2) and Hochiminh City Power Corporation (CG3) have been identified as the second and third most prominent options, respectively. According to the data shown in Table 15 and Fig 4, Power Engineering Consulting JSC3 (CG6) is identified as the least favorable option in terms of corporate sustainable growth, as it received the lowest appraisal score.

### 5.2. Discussions

The authors used the MCDM model based on SF-AHP to evaluate the priority of main criteria and sub-criteria to assess the efficacy of sustainable business development management serving state-owned electricity enterprises in Vietnam. The results showed that the main dimensions of state-owned electricity enterprises in Vietnam evaluation criteria are ranked as follows: Business Management, Corporate Social Responsibility, and Corporate Governance Shareholder, respectively. The responsibility of supervising the organization, coordination, and implementation of diverse business operations constitutes business management. This may involve overseeing various business operations, such as marketing, sales, and accounting [76]. In order to stay in business, customers, businesses, and governments all over the world have come to realize how important it is to use sustainable business practices [77]. Corporate social responsibility (CSR) refers to the corporation’s decision-making process regarding its operations within the social, political, legal, and ethical frameworks of its surroundings. Therefore, a company’s CSR strategy is closely conected to its fundamental value propositions to its customers, employees, suppliers, shareholders, and other important stakeholders [78]. For sustainable corporate governance, it is impossible not to mention factors related to shareholders. A significant function of shareholders is to ensure good corporate governance. They have a pecuniary stake in the organization’s performance as co-owners. In addition to risk management, they might be concerned with the social, environmental, and economic impacts of the organization. As their interests affect the majority of a business’s operations, shareholders are vital to its performance and profitability.

Upon further examination of Table 11, as indicated by the local weight ranking scale measure, it becomes obvious that strategic planning is regarded as the most significant aspect of business management. Strategic planning facilitates the alignment of mission, goals, strengths, opportunities, priorities, and assessment in a way that enhances decision-making. Strategic planning results in the implementation of strategic management, which provides guidance, leadership, and orientation for the organization [79]. Our findings align with Tapera’s research [80], which elucidates the fundamental components of strategic management: strategic vision, objectives, strategy creation, strategy implementation, evaluation, and corrective action initiation. The research also examines the corporate governance component of strategic management. In order to enhance corporate governance and foster sustainable development, it is imperative to expeditiously execute effective policies and practices. For instance, allocating sufficient financial and human resources is crucial for ensuring successful implementation, designating a team accountable for implementation, organizing periodic review meetings, closely monitoring key performance indicators, and remaining adaptable and flexible are all essential components of such practices [81]. Furthermore, the corporate social responsibility aspect considers quality as the most important factor. The result match with some previous studies [55, 82]. Companies gain consumer loyalty, brand awareness, and cost control with product quality. Customers buy more from trusted organizations, and product quality disputes can reduce product returns, problems, and losses. Therefore, in order to sustain their reputation as an industry leader with high-quality products, businesses should routinely reevaluate the quality of their present products, enhance production procedures, increase staff capacity, upgrade technology, and develop and improve the quality control system [83]. The responsibility of the board is the most important task of the Corporate Governance Shareholder aspect. The board represents the shareholders and is responsible for making comprehensive policy decisions and ensuring supervision. The board of directors is entrusted with a fiduciary obligation towards the shareholders. This entails the board’s financial and other obligations to ensure the business operates efficiently, thereby safeguarding the shareholders from financial losses. Hence, organizations should determine a governance framework, foster a culture of trust and collaboration, engage stakeholders and solicit their feedback, recognize diversity and inclusion, leverage technology and data to implement strategies and monitoring mechanisms [84].

In general, based on Table 11’s results, the most prominent criterion in corporate governance was found to be concerns related to business mmanagement. SOEs should focus enhancing customer satisfaction and continuously improve the impacts of strategic planning. The next crucial aspect in corporate governance was the implementation of effective corporate social responsibility. Managers should collaborate with standard-setting authorities and stakeholders to address any uncertainties in governance regulations to decrease the amount of potentially harmful waste. Managers must prioritize fostering community engagement and tackling inequality concerns as they are crucial. Corporate governance shareholder factors have a significantly lower level of influence, although they nonetheless remain significant. Ensuring the preservation of shareholders’ rights and obligations is crucial for managers. The suggested MCDM framework provides managers with a reliable instrument to compare the performance of corporate governance with that of rivals and identify specific areas that need improvement.

According to the EDAS method, the sustainable development of the three business organizations designated as the most efficient among state-owned electricity enterprises in Vietnam are Northern Power Corporation Contact Center, Southern Power Corporation, Hanoi City Power Corporation. In fact, the Northern Power Corporation implements all its activities with sensitivity and strives to streamline CSR interventions in areas that have significant impact, both in terms of quality and scale. The key domains of intervention encompass livelihood, education, employability, empowerment, health, access to potable water, sanitation, sports, and the development of rural infrastructure. These endeavors are supported by measures promoting renewable energy and other programs aimed at environmental protection, along with the national goal on Swachh Bharat, School toilets, and Skill India initiatives [85]. Southern Power Corporation is advocating for the implementation of digital technology to revolutionize and enhance the administration and operation procedures of the power system. Enhancing operational procedures, optimizing and strengthening corporate governance to facilitate data-driven decision making. An impressive achievement is the effective deployment of Vietnam Electricity’s interconnected and collaborative applications, following a predetermined plan, alongside the development of tailored software to fulfill the needs of Southern Power Corporation’s sustainable growth [86]. In addition to power quality and services, Hochiminh City Power Corporation has innovated, enhanced corporate governance, and developed partnerships with local and global partners in recent years. Promote business culture and social initiatives for community benefit and sustainable growth. The current sustainable growth process of Hochiminh City Power Corporation requires more comprehensive and synchronized solutions [87].

## 6. Conclusions, limitations, and future work

### 6.1. Conclusions

This study presents an innovative methodology that integrates the SF-AHP and EDAS techniques. A panel of twelve electricity industry experts, representing both public and private sectors, is assembled to provide input on the relative importance of criteria and assess the sustainable development efforts of electricity enterprises in Vietnam. The evaluation framework consists of the SF-AHP for determining the significance of evaluation criteria and the EDAS model for ranking companies according to their sustainable development management. This two-stage MCDM model employs exhaustive criteria based on sustainable growth and factors identified by the leading electric power industry in Vietnam. The study reveals that in regard to sustainable development decisions made by corporations, business management and corporate social responsibility considerations take precedence over shareholder and corporate governance factors. The examination of sub-criteria weights indicates that process management factors, board responsibilities, strategic planning, leadership, shareholder rights, and responsibility for sustainable development have a substantial impact on management decisions regarding sustainable development. According to the EDAS analysis, the three leading organizations in the field of sustainable development management are Hanoi City Power Corporation, Northern Power Corporation Contact Center, and Southern Power Corporation. By employing the proposed MCDM methods in a practical case study, the efficacy of the proposed method is validated. The results of this study provide valuable insights for institutional managers and policy makers who advocate for well-informed management decisions. The proposed method’s robustness is demonstrated by the continuous priority order of the top companies across various MCDM approaches.

The research’s contributions to the existing body of literature and practical application can be defined as follows.

1. This study is the first attempt to examine the management practices of state-owned electric power firms in Vietnam with regards to corporate sustainable development. The analysis used a unique combination of the DEA, SF-AHP, and EDAS models.
2. A set of key factors related to sustainable development management in Vietnamese SOEs are identified and categorized. A case study from the Vietnamese electric power sector is used to validate the suggested models.
3. For expert evaluations on a broader linguistic scale, the weighting of the criterion is computed using spherical fuzzy sets; this emulates the decision-making process in environments rife with uncertainty. The relative weights are computed by SF-AHP, while the EDAS model possesses the capability and precision to rank the enterprises.
4. The suggested hybrid decision-making approach can serve as a framework for Vietnamese state-owned enterprises to enhance corporate governance of their operational practices toward sustainable growth.

### 6.2. Limitations, and future works

Considering its contributions, this work possesses certain limitations that warrant consideration in future research. First, utilizing a specific case study method may limit the applicability of the results. Performing numerous case studies across various industries would augment the practicality and credibility of the concept. Second, the emphasis on the electric power industry implies that the conclusions may not be readily applicable to other industries. Future research should investigate the efficacy of the model across different sectors to expand its range. Third, the dependence on expert judgments presupposes their precision and dependability. Conducting sensitivity analysis would provide useful insights into the model’s robustness and its vulnerability to expert judgments. Fourth, there are other MCDM frameworks that utilize spherical fuzzy, such as the spherical fuzzy Technique for Order Preference Similarity Ideal Solution (SF-TOPSIS) method and application of the Spherical Fuzzy Analytic Network Process (SF-ANP) allows for the assessment of sustainable development criteria, the obtained outcomes may then be compared to the findings of this study. Finally, a number of sub-criteria on social responsibility, environmental sustainability, and stakeholder engagement are included in this study. However, these sub-criteria are not enought to reflect all relevant aspects. Therefore, future studies need to consider carefully more of these criteria to capture the specific challenges and opportunities that SOEs in Vietnam face in the future. Toenhance the model’s efficacy and practical applicability, it is important for future research to tackle these limitations. Researchers can enhance the comprehension and practicality of the suggested model for guiding sustainable development management decisions by performing several case studies across different industries, examining the model’s sensitivity, and broadening the evaluation criteria.

## Supporting information

S1 Dataset (ZIP)
